# Supplementary material for: Empirical methods for controlling false positives and estimating confidence in ChIP-Seq peaks
Source: BMC Bioinformatics. 2008 Dec 5;9:523. doi: 10.1186/1471-2105-9-523 (PMC2628906; doi:10.1186/1471-2105-9-523)
Supplement: Additional File 1 — A variety of html documents from the USeq web site detailing the available applications, their best usage, output file type descriptions, command line menus, etc. [file 1471-2105-9-523-S1.zip › USeqUserGuides/JavaDoc.zip!/JavaDoc/meme/MemeMotif.html]

MemeMotif


|  |  |  |
| --- | --- | --- |
| **Overview** \| **Package** \| **Class** \| **Use** \| **Tree** \| **Deprecated** \| **Index** \| **Help** | |  |
| PREV CLASS   **NEXT CLASS** | **FRAMES**    **NO FRAMES**     **All Classes** |  |
| SUMMARY: NESTED \| FIELD \| CONSTR \| METHOD | DETAIL: FIELD \| CONSTR \| METHOD |  |


---


## meme Class MemeMotif

```
java.lang.Object
  meme.MemeMotif
```

---

``` public class MemeMotif extends java.lang.Object ```

This contains info generated from parsing a meme report, each found motif is represented by this class.

---

**Constructor Summary**

`MemeMotif()`


| **Method Summary** | |
| --- | --- |
| `double[][]` | `getAdd1PSPM()` |
| `int[][]` | `getFreqMatrix()` |
| `java.lang.String[]` | `getHits()` |
| `double[][]` | `getLLAdd1PSPM(double pA, double pC, double pG, double pT)` |
| `double[][]` | `getLLMemePSPM(double pA, double pC, double pG, double pT)` |
| `MemeParser` | `getMemeParser()` |
| `double[][]` | `getMemePSPM()` |
| `java.lang.String` | `getMotifDesc()` |
| `java.lang.String` | `getMotifSites()` |
| `java.lang.String` | `getMotifSumLn()` |
| `int` | `getNumberName()` |
| `static double[][]` | `makeAddOnePSPM(java.lang.String[] hits)`             This method returns a position specific probability weight matrix given an array of Strings, each of the same size, containing GATC, no gaps, no N's. |
| `static int[][]` | `makeFrequencyMatrix(java.lang.String[] hits)`             Generates a matrix of the number of As Cc Gg Ts (top) by 1,2,3,4...positions in the motif (side) observed in all the Strings of the String[]. |
| `static double[][]` | `makeLLPSPM(double[][] PSPM, double pA, double pC, double pG, double pT)`             takes a double[][] where array[0] refers to the first position in a motif array[0][0 to 3] = probability of A, C, G, or T nucleotide at that position. |
| `static double[][]` | `makePSPM(java.lang.String[] hits)`             This method returns a position specific probability weight matrix given an array of Strings, each of the same size, containing GATC, no gaps, no N's. |
| `void` | `setHits(java.lang.String[] x)` |
| `void` | `setMemeParserRef(MemeParser x)` |
| `void` | `setMemePSPM(double[][] x)` |
| `void` | `setMotifDesc(java.lang.String x)` |
| `void` | `setMotifSites(java.lang.String x)` |
| `void` | `setMotifSumLn(java.lang.String x)` |
| `void` | `setNumberName(int x)` |
| `java.lang.String` | `toString()` |

**Methods inherited from class java.lang.Object**

`equals, getClass, hashCode, notify, notifyAll, wait, wait, wait`

**Constructor Detail**

### MemeMotif

```
public MemeMotif()
```


**Method Detail**

### getNumberName

```
public int getNumberName()
```

---


### getMotifSumLn

```
public java.lang.String getMotifSumLn()
```

---


### getMotifDesc

```
public java.lang.String getMotifDesc()
```

---


### getMotifSites

```
public java.lang.String getMotifSites()
```

---


### getHits

```
public java.lang.String[] getHits()
```

---


### getMemePSPM

```
public double[][] getMemePSPM()
```

---


### getLLMemePSPM

```
public double[][] getLLMemePSPM(double pA,
                                double pC,
                                double pG,
                                double pT)
```

---


### getAdd1PSPM

```
public double[][] getAdd1PSPM()
```

---


### getLLAdd1PSPM

```
public double[][] getLLAdd1PSPM(double pA,
                                double pC,
                                double pG,
                                double pT)
```

---


### getMemeParser

```
public MemeParser getMemeParser()
```

---


### setNumberName

```
public void setNumberName(int x)
```

---


### setMotifSumLn

```
public void setMotifSumLn(java.lang.String x)
```

---


### setMotifDesc

```
public void setMotifDesc(java.lang.String x)
```

---


### setMotifSites

```
public void setMotifSites(java.lang.String x)
```

---


### setHits

```
public void setHits(java.lang.String[] x)
```

---


### setMemePSPM

```
public void setMemePSPM(double[][] x)
```

---


### setMemeParserRef

```
public void setMemeParserRef(MemeParser x)
```

---


### makeLLPSPM

```
public static double[][] makeLLPSPM(double[][] PSPM,
                                    double pA,
                                    double pC,
                                    double pG,
                                    double pT)
```

:   takes a double[][] where array[0] refers to the first position in a motif
    array[0][0 to 3] = probability of A, C, G, or T nucleotide at that position.
    MakeLLPSPM converts each nucleotide probablility to a log likelihood value by
    taking the natural log of the probability ration ie array[0][0]/ pA .
    pN is background probability of a particular nucleotide (ie 0.25)

---


### toString

```
public java.lang.String toString()
```

:   **Overrides:**: `toString` in class `java.lang.Object`

---


### makeAddOnePSPM

```
public static double[][] makeAddOnePSPM(java.lang.String[] hits)
```

:   This method returns a position specific probability weight matrix given an array
    of Strings, each of the same size, containing GATC, no gaps, no N's.
    The weight matrix is an Add One matrix where a single psuedo count is added to
    all base counts when a zero is found in a particular base bin.
    The order of the array is ACGT for each position in a single hit.

---


### makePSPM

```
public static double[][] makePSPM(java.lang.String[] hits)
```

:   This method returns a position specific probability weight matrix given an array
    of Strings, each of the same size, containing GATC, no gaps, no N's.
    The order of the array is ACGT for each position in a single hit.

---


### makeFrequencyMatrix

```
public static int[][] makeFrequencyMatrix(java.lang.String[] hits)
```

:   Generates a matrix of the number of As Cc Gg Ts (top) by 1,2,3,4...positions in the
    motif (side) observed
    in all the Strings of the String[]. The String[] should contain equal length Strings,
    comprised entirely of GATC.

---


### getFreqMatrix

```
public int[][] getFreqMatrix()
```


---


|  |  |  |
| --- | --- | --- |
| **Overview** \| **Package** \| **Class** \| **Use** \| **Tree** \| **Deprecated** \| **Index** \| **Help** | |  |
| PREV CLASS   **NEXT CLASS** | **FRAMES**    **NO FRAMES**     **All Classes** |  |
| SUMMARY: NESTED \| FIELD \| CONSTR \| METHOD | DETAIL: FIELD \| CONSTR \| METHOD |  |


---
